# Enhanced Platinum-Based Thin-Film Catalysts for Electro-Oxidation of Methanol

**DOI:** 10.3390/ma17225575

**Published:** 2024-11-15

**Authors:** Dušan V. Tripković, Dragana L. Milošević, Sanja I. Stevanović, Ksenija Dj. Popović, Vladislava M. Jovanović

**Affiliations:** 1 Department of Electrochemistry, Institute of Chemistry, Technology and Metallurgy, University of Belgrade, Njegoševa 12, 11000 Belgrade, Serbia; sanjas@ihtm.bg.ac.rs (S.I.S.); ksenija.pop@sbb.rs (K.D.P.); vlad@tmf.bg.ac.rs (V.M.J.); 2 Department of Ecology and TechnoEconomics, Institute of Chemistry, Technology and Metallurgy, University of Belgrade, Njegoševa 12, 11000 Belgrade, Serbia; dragana.milosevic@ihtm.bg.ac.rs

**Keywords:** methanol electro-oxidation, thin-film catalysts, surface reconstruction, metal–support interactions, enhanced catalytic properties

## Abstract

Surface morphology is one of the critical factors affecting the performance of electrocatalysts. Thus, with careful manipulation of the surface structures at the atomic level, the effectiveness of the catalyst can be significantly improved. Heat treatment is an effective method for inducing surface atom rearrangement, hence modifying the catalyst’s characteristics. This study investigated the substrate’s influence and the effect of thermal annealing on the morphology and surface reconstruction of platinum (Pt) thin-film catalysts. Our findings indicate that heat treatment in a reductive atmosphere (95% Ar + 5% H_2_) at 300 °C can significantly impact the degree of rearrangement of surface atoms. This process induces long-range ordering, resulting in domains with a high proportion of (111) and (100) sites without an epitaxial template. Considering that the reactivity of low-index platinum single crystals for the methanol oxidation reaction follows the following sequence Pt(111) < Pt(110) < Pt(100), increasing the proportion of (100) planes leads to a notable enhancement (up to three times) in performance, compared to untreated catalysts. Furthermore, considering the amount of precious metal consumed, a mass-specific current density obtained on annealed Pt@Ni is larger by one order of magnitude and ~2 times that obtained on Pt@Cr and Pt@GC_ox_ catalysts, respectively. Our results demonstrate that an easy-to-implement way of controlling atomic orientations improves catalyst performance. With this contribution, we propose a method for designing improved electrocatalysts, as catalytic reactions occur only at the surface.

## 1. Introduction

Oxidation of methanol on platinum (Pt) is one of the most studied reactions due to the possible commercialization of direct methanol fuel cells (DMFC) [1,2,3,4,5,6]. One of the leading technical challenges preventing the use of methanol fuel cells in commercial devices is the sensitivity of the reaction to self-poisoning, in addition to the high cost and limited supplies of Pt [7,8]. Although the dehydrogenation of methanol in sequential steps implies the existence of many adsorbates, spectroscopic methods conducted in situ on Pt single crystals have revealed only CO_ad_ species present at the surface [9,10]. Adsorbed CO was proposed as a reactive intermediate in methanol oxidation [11,12], but considering the fact that it partially blocks the surface at low potentials, it can be regarded as a poison too. Namely, the oxidation of CO_ad_ is considered a so-called “fingerprint” of the surface of the Pt electrode because the surface structure of platinum is directly related to the kinetics of this reaction. By electrochemical examination of this reaction, we can obtain information about the structure of the catalyst surface. It is well known that the oxidation of CO_ad_ follows the Langmuir–Hinshelwood mechanism [7]. In the case of an acidic electrolyte, pre-oxidation and main oxidation areas are distinguished. In the pre-oxidation area, non-competitive adsorption occurs between CO and OH species, while in the main oxidation area, their adsorption becomes competitive. The degree of coverage of the adsorbed CO and OH species changes with the change in potential; i.e., it is inversely proportional to the potential. CO is predominantly adsorbed on terraces of single-crystal planes, while OH species are on defects and steps. For this reason, the oxidation of CO occurs on defects and step sites [13]. Adsorbed OH^−^ generated from oxidative water decomposition in an acid solution is suggested as the second active intermediate [11,14]. Water dissociation and OH formation on highly ordered planes of platinum are possible only at very positive potential values (0.85 vs. RHE), which is the reason for the rapid removal of CO near defects and steps where OH species are located. Herrero and coworkers [15] also confirmed that the electro-oxidation of methanol takes place only in the presence of adsorbed OH. Their results, obtained on the three basal planes of platinum, confirm that adsorbed OH is involved in the mechanism beyond providing the oxygen group required to oxidize adsorbed CO. Adsorbed OH has high mobility on the surface and the favorable shift of hydrogen from the hydroxy group of methanol to adsorbed OH results in the immediate activation of methanol. As soon as the methanol comes into contact with the surface, the favorable interaction with adsorbed OH produces adsorbed methoxy [15]. Thus, theoretically, the efficiency of Pt-based catalysts could be enhanced by the well-balanced coadsorption of OH^−^ anions and methanol. Generally, electrochemical reactions that proceed through the surface adsorption of intermediates usually display structure sensitivity.

Studies on Pt low-index single crystals have confirmed that the electrochemical oxidation of methanol is indeed a structure-sensitive reaction [13,16,17,18,19]. Using on-line electrochemical mass spectrometry (OLEMS), Housmans et al. found that the maximum reactivity of the methanol oxidation reaction on low-index Pt single crystals increases in the following order Pt(111) < Pt(110) < Pt(100) [20].

Since catalytic reactions occur on the active material surface, one of the efficient ways to reduce the amount of Pt and bridge the gap between well-established single-crystal surfaces and real systems is to produce a catalyst in the form of a thin film. These films are ideal for investigations of electrochemical processes in fuel cells, and, in addition, they can provide insight into the link between bulk surfaces and nanoscale systems, serving as templates for developing commercial electrocatalysts. Pt deposited on carbon material is the most common PEMFC, offering an extensive surface area, good electrical conductivity, and an appropriate structure [21]. However, carbon support materials usually suffer from degradation under severe PEMFC operating conditions [22,23]. Consequently, carbon corrosion decreases the electrochemical surface area (ECSA) and affects the stability and durability of catalysts, which hinders long-term operations [24,25]. Therefore, electrochemically stable support materials must be carefully chosen [26,27]. Research has shown that interactions between the deposited metal and the support play an essential role in determining the overall properties of the catalyst [28,29,30]. To investigate whether it is possible to control structural changes using thermal treatment, Pt catalysts in the shape of thin films over glassy carbon (GC), Ni, and Cr were studied. Metal support in the overall catalyst would provide better catalyst properties due to Ni and Cr thermal stability compared to GC [31,32,33]. Finally, metal–support solid interactions (SMSIs) can significantly influence a catalyst’s activity, stability, and selectivity due to electronic, geometric, and bifunctional effects.

## 2. Materials and Methods

### 2.1. Electrode Preparation

High-purity Ni and Cr disks, along with a glassy carbon electrode (GC) (Sigradur-Sigri, Elektrographite, GmbH, Bocholt, Germany), all 6 mm in diameter, served as substrates for obtaining thin-film catalysts. Before each electrochemical experiment, all electrodes were mirror-polished using silicon carbide grinding paper (Buehler, P800–P4000) and 1–0.05 µm alumina (Buehler, Chipping Norton, NSW, Australia). After polishing, the surface was sonicated for 2–3 min and rinsed with high-purity water (“Millipore”, 18.2 MΩcm resistivity, Burlington, MA, USA).

Polished GC electrodes were electrochemically treated by anodic polarization in 0.1 M HClO_4_ at 2.0 V vs. reversible hydrogen electrode (RHE) for 190 s. We designated the activated glassy carbon surfaces as GCox.

Before depositing the Pt, a cyclic voltammogram of GC_ox_ and pure Ni or Cr electrode was recorded. The potential range was 0.045 V to 1.245 V vs. RHE sweep rate 50 mV s^−1^ in a 0.1 M HClO_4_ supporting solution. This treatment was performed to ensure that the surfaces were clean and free of Pt left behind from previous experiments.

The electrodeposition of Pt on GC_ox_, Ni, or Cr substrates (S in further text) was under potentiostatic conditions in a 0.1 M HClO_4_ + 1 mM H_2_PtCl_6_ solution previously deoxygenated with N_2_ gas. The deposition was performed in three successive steps, from an initial potential hold at 0.045 V vs. RHE for 2 s to a final potential of 0.065 V vs. RHE for 50 s. The quantity of Pt was calculated based on the integrated charge obtained from the i-t transient response, considering the correction for substrate charging. This method results in the deposition of stable Pt thin films with a thickness of approximately 100 monolayers of Pt. After the deposition, the as-prepared Pt@S electrodes were cycled until a stable voltammogram was achieved and then annealed in a reductive atmosphere (95% Ar + 5% H_2_) at 300 °C for 2 h [31].

### 2.2. Characterization of the Catalysts

Catalyst surface morphology was probed using atomic force microscopy (AFM) with a NanoScope III D (Veeco, Plainview, NY, USA) instrument that was operated in taping mode under ambient conditions. Silicon nitride cantilevers with a force constant of 0.06 N m^−1^ were used. Based on AFM images, all surfaces were analyzed by observing five different areas of the samples. AFM measurements were performed before and after each thermal treatment.

### 2.3. Electrochemical Measurements

All electrochemical experiments were carried out in a 0.1 M HClO_4_ electrolyte at room temperature in a standard three-electrode compartment glass cell. Pt wire was used as the counter and a bridged SCE as a reference electrode. The electrolytes were prepared with p.a. grade chemicals (“Merck”, Frenchs Forest NSW, Australia) and high-purity water. All solutions were pre-saturated with nitrogen and kept under an inert atmosphere during the experiments.

After Pt deposition, the electrodes were carefully washed with high-purity water and transferred to the cell with 0.1 M HClO_4_ electrolyte. The electrochemical surface area of Pt@S electrodes was calculated from the charge in the hydrogen adsorption/desorption region (potential range from −0.2 V to 0.2 V) with a correction for double-layer charging. This calculation was based on the steady-state cyclic voltammograms in the supporting electrolyte, assuming a 210 μC/cm^2^ charge for a monolayer of adsorbed hydrogen.

The specific surface orientation on Pt@S samples was studied by means of irreversible Bi adsorption, in order to obtain relevant information regarding (111) domains [31,34] and oxidation of ammonia (0.3 M in 0.1 M NaOH at a sweep rate of 50 mV s^−1^) that was utilized to confirm the presence of (100) planes.

The Pt@S electrode’s electrocatalytic activity for methanol oxidation was assessed in a 0.1 M HClO_4_ solution with the addition of 0.5 M CH_3_OH to the supporting electrolyte while maintaining the electrode potential at −0.2 V and the sweep rate at 50 mV s^−1^.

## 3. Results and Discussion

### 3.1. AFM Surface Characterization

**Figure 1:**
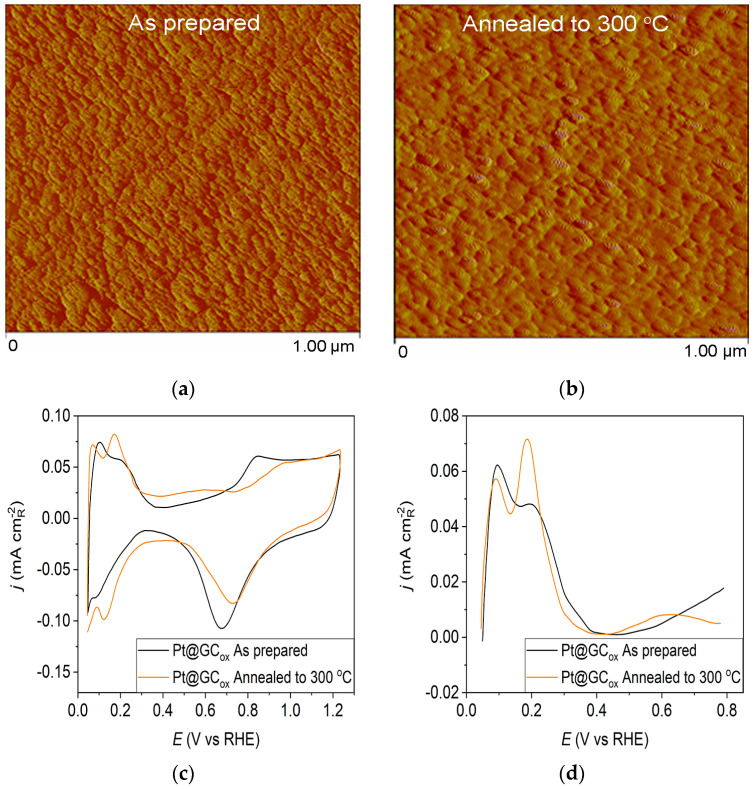

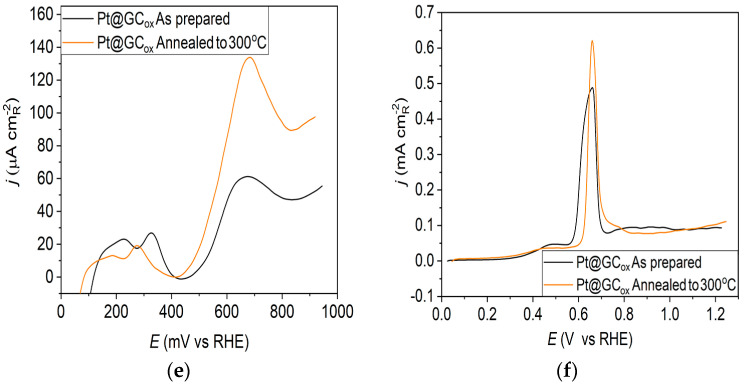
**Pt@GC_ox_ catalyst**: (**a**) AFM images (1 × 1 µm)—as-prepared; (**b**) AFM images (1 × 1 µm)—annealed to 300 °C; (**c**) voltammograms; (**d**) forward scan after double layer subtraction; (**e**) the oxidation of ammonia for determining the contribution of 100 planes; (**f**) CO stripping curves. Black curves—as-prepared; orange curves—annealed to 300 °C. Test electrolyte 0.1 M HClO_4_; sweep rate: 50 mV s^−1^.

**Figure 2:**
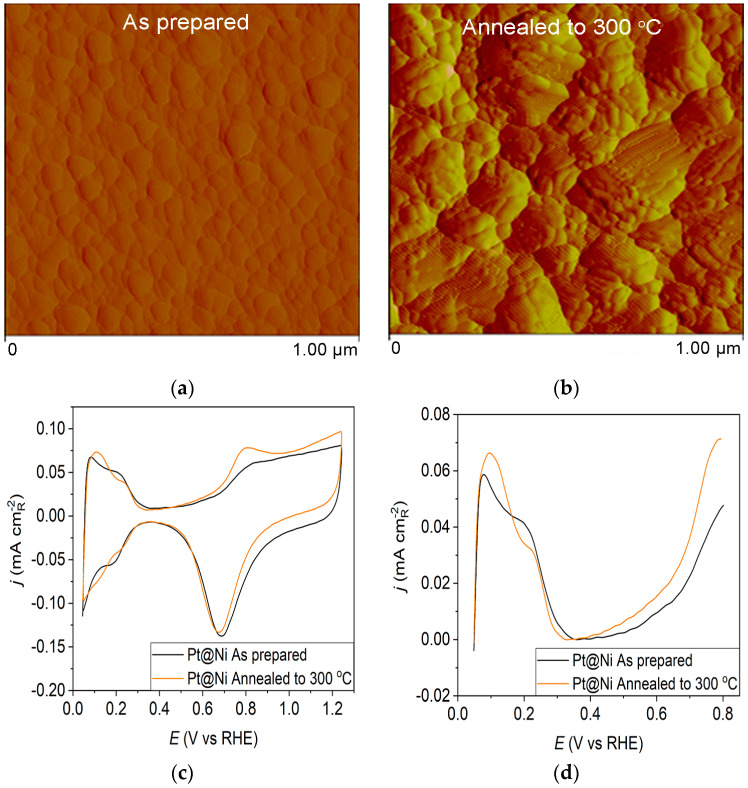

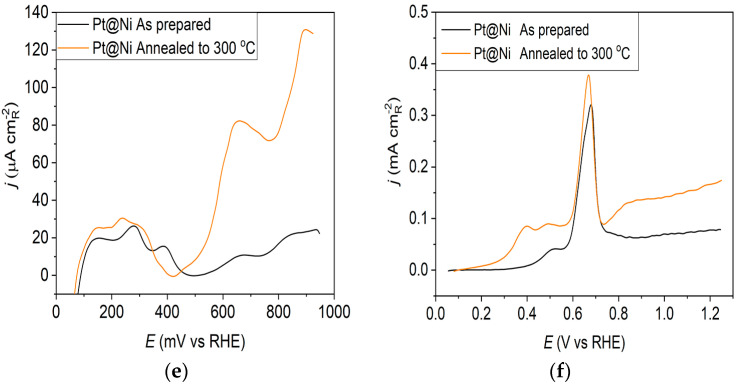
Pt@Ni catalyst: (**a**) AFM images (1 × 1 µm)—as-prepared; (**b**) AFM images (1 × 1 µm)—annealed to 300 °C; (**c**) voltammograms; (**d**) forward scan after subtraction of double layer; (**e**) the oxidation of ammonia for determining the contribution of 100 planes; (**f**) CO stripping curves. Black curves—as-prepared; orange curves—annealed to 300 °C. Test electrolyte 0.1 M HClO_4_; sweep rate: 50 mV s^−1^.

**Figure 3:**
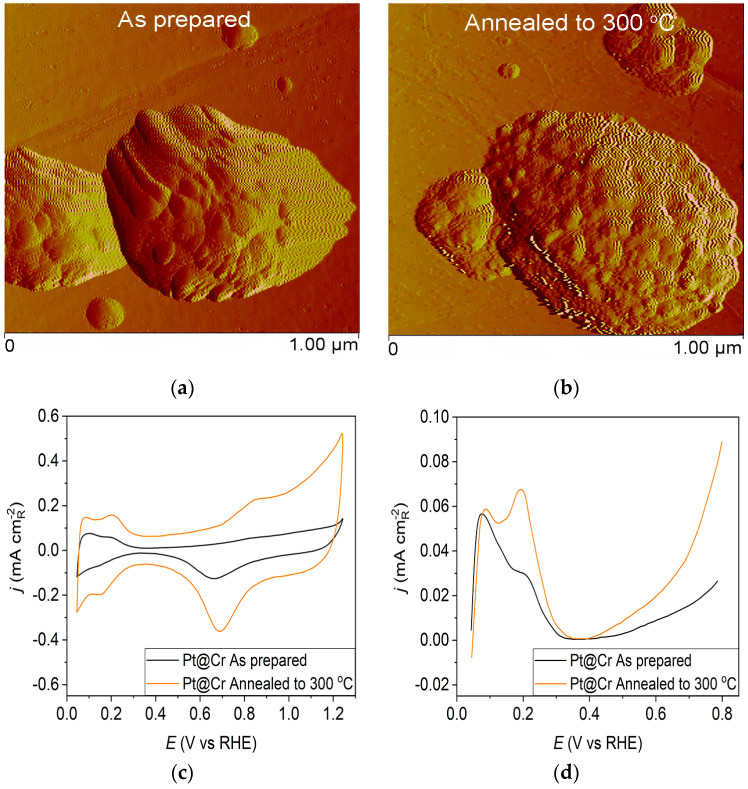

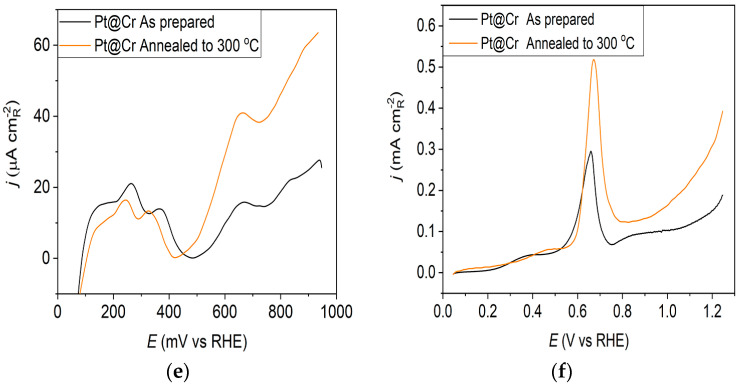
Pt@Cr catalyst: (**a**) AFM images (1 × 1 µm)—as-prepared; (**b**) AFM images (1 × 1 µm)—annealed to 300 °C; (**c**) voltammograms; (**d**) forward scan after double layer subtraction; (**e**) the oxidation of ammonia for determining the contribution of 100 planes; (**f**) CO stripping curves. Black curves—as-prepared; orange curves—annealed to 300 °C. Test electrolyte 0.1 M HClO_4_; sweep rate: 50 mV s^−1^.

The AFM imaging technique was used to assess surface morphology, and the results are summarized in Figure 1, Figure 2 and Figure 3. Figure 1a displays the morphology of the as-prepared Pt@GC_ox_ electrode, which consists of small Pt clusters with relatively uniform sizes due to the homogeneity of the deposition process. Following annealing, the AFM image of the thermally treated Pt@GC_ox_ electrode (Figure 1b) illustrates that the controlled thermal treatment has led to the migration and agglomeration of Pt particles, producing larger clusters [31].

The as-prepared Pt@Ni electrode (Figure 2a) contains spherical clusters, equally sized and uniformly distributed all over the surface. The AFM image of the Pt@Ni electrode obtained after annealing (Figure 2b) illustrates that the controlled thermal treatment has led to the migration of Pt particles and creates approximately two times larger clusters than those in the as-prepared sample.

However, the as-prepared Pt@Cr electrode (Figure 3a) possesses predominantly large, spherical-shaped Pt agglomerates. The polishing lines from the underlying substrate can also be seen, concluding that some parts of the substrate remain uncovered by the Pt deposit. The agglomeration effect could also be observed for the Pt@Cr electrode annealed to 300 °C (Figure 3b). The agglomerates are slightly larger in diameter, but the shape remains unchanged compared to the as-prepared sample.

### 3.2. Electrochemical Characterization

Pt@S catalysts have been studied using the potentiodynamic method. Figure 1c, Figure 2c and Figure 3c show the steady-state voltammograms of the three Pt-based thin-film catalysts in perchloric acid solution, obtained before and after annealing in H_2_/Ar at 300 °C. The presented voltammetric curves show that the processes during the potential sweep can be divided into hydrogen adsorption/desorption in the potential region up to E~0.35 V, parallel with the reversible OH^−^ adsorption up to E~0.75 V; later, at more positive potentials, the irreversible oxide formation occurs. All three electrodes show comparable behavior in the reactions associated with H^+^ and OH^−^ adsorption/desorption. At higher potentials, the Pt@GC_ox_ electrode shows a slower onset of oxide formation and a shift in the peak corresponding to oxide reduction towards more positive potentials. It indicates weaker adsorption of oxide species compared to the other two electrodes. The voltammetric profile of the as-prepared Pt@S electrodes (Figure 1d, Figure 2d and Figure 3d—black lines) in the hydrogen area (0.05 V < E < 0.4 V) looks similar to the voltammetric profile for the polycrystalline Pt electrode [31,35,36] and corresponds directly to the domains with a particular surface orientation such as nature and surface sites density. Therefore, the voltammograms display adsorption states connected with (110) and (100) sites at 0.075 V and 0.195 V, respectively. The most intense peak is at 0.075 V, with a shoulder appearing at 0.195 V. Additionally, a minor shoulder at 0.295 V is hardly detectable, which is typical for a small amount of brief (100) terraces [37,38,39].

Voltammograms for the Pt@S electrodes annealed in the reductive atmosphere to 300 °C are shown in Figure 1c, Figure 2c and Figure 3c—orange lines. The heat treatment changes the shape of the voltammogram in the hydrogen and oxide regions, as demonstrated by comparing the presented curves. For the Pt@GC_ox_ electrode (Figure 1d—orange curve), the main characteristic of the voltammogram is a very sharp peak at 0.195 V, which corresponds with the (100) edge and the corners of the Pt surface sites. A wide peak at ~0.55 V shows the presence of a considerable number of domains with the (111) orientation, in contrast to the as-prepared electrode (Figure 1d—black curve) where it is not detectable. This property directly links to the existence of two-dimensional ordered (111) domains. The (111) sites also have influence through the steps at the junction (111) × (111) ≡ (110) sites that contribute to the state at 0.075 V. Comparable sharp peaks are seen on stepped surfaces adjacent to Pt (111) [35,40].

For the Pt@Ni electrode annealed at 300 °C (Figure 2c—orange curve), the voltammogram is very similar to the as-prepared one (Figure 2c—black curve) in the hydrogen adsorption zone. Still, it demonstrates an increase in current in the oxide region. The increase in peak current at 0.075 V might indicate that thermal annealing causes changes in the specific surface structures because of the rearrangement of atoms on the surface in favor of the (110) plane. The peak at 0.8 V, which results from the adsorption of OH^−^ anions at the surface, shows the same growth trend upon annealing, indicating an increase in surface oxophilicity.

The Pt@Cr electrode annealed in H_2_/Ar to 300 °C (Figure 3d—orange curve) displays an increase in current at 0.195 V compared to the voltammogram for the as-prepared catalyst (Figure 3d—black curve). This feature is characteristic of hydrogen adsorption at (100) step sites on (111) terraces with a contribution from (100) steps near (100) terraces for the same potential [19].

### 3.3. Surface Orientation

The irreversible adsorption of bismuth, which selectively occurs at the Pt(111) surface sites, might provide helpful information on the ordered domains [36,37]. The selective adsorption of Bi shows that with electrode annealing to 300 °C, the fraction of Pt(111) terrace sites increases 2.3, 3.0, and 4.7 times on Pt@GC_ox_, Pt@Ni, and Pt@Cr, respectively, relative to the as-prepared ones (Table 1) [41,42].

Ammonia oxidation on Pt surfaces is a structure-sensitive reaction that occurs nearly exclusively on (100) sites. It is also susceptible to the width of the (100) terraces and the density of steps with (100) symmetry [40]. The results obtained for ammonia oxidation in alkaline solution shown in Figure 1e, Figure 2e and Figure 3e display higher activity for as-prepared Pt@GC_ox_ (Figure 1e) compared to Pt@Ni (Figure 2e) and Pt@Cr (Figure 3e) catalysts, indicating that the most significant number of (100) domains is present at the Pt@GC_ox_ electrode. The mild annealing conditions to 300 °C can be sufficient to induce surface faceting that assumes a higher number of (100) sites, as evidenced by the increase in ammonia oxidation activity on all tested surfaces. The results show that annealing at 300 °C increases the number of sites with (100) orientation for 2.0, 4.0, and 5.3 times on Pt@GC_ox_, Pt@Ni, and Pt@Cr electrodes, respectively, relative to the as-prepared ones.

Bi adsorption and ammonia oxidation findings show that even gentle annealing conditions are sufficient to cause surface faceting. We successfully increased the ratio of both (111) and (100) sites through thermal treatment in a reductive atmosphere. Additionally, we recognized that the majority of (110)-like sites can be considered as the sum of the remaining sites not related to either (111) or (100) directions.

The oxidation of CO_ads_ was employed to characterize the Pt@S catalysts (Figure 1f, Figure 2f and Figure 3f) due to its remarkable sensitivity to specific surface structures [19,38,43,44]. However, one should be careful when concluding the CO stripping experiments since the substrate can significantly affect the CO oxidation currents. For this reason, oxidation of pre-adsorbed CO was used to confirm the results obtained from the irreversible adsorption of Bi and ammonia oxidation.

The stripping voltammograms recorded on all electrodes display the typical features characteristic of CO oxidation [45,46], (Figure 1f, Figure 2f and Figure 3f). It is important to mention that the backward and the second positive-going scans are identical to the voltammograms for the surfaces free of CO_ad_.

All as-prepared electrodes (Pt@GC_ox_, Pt@Ni, and Pt@Cr) possess a very similar voltammetric profile with the main oxidation peak at approximately the same potential (~0.67 V), indicating that the reaction takes place at the same active sites on all three catalysts. Due to the deposition process and the nature of each substrate, the active surface area is not always the same, ultimately producing a difference in the recorded peak intensities. The pre-peak shoulder observed from 0.25 to 0.57 V is attributed to the removal of CO_ad_ from domains containing surface defects, where the oxidation of adsorbed CO begins at lower potentials [13]. As can be seen on Figure 3f, the oxidation of CO_ad_ starts at the most negative potential on the as-prepared Pt@Cr electrode, followed by the Pt@GC_ox_ (Figure 1f) and Pt@Ni catalysts (Figure 2f). Figure 1f, Figure 2f and Figure 3f show the CO_ad_ stripping voltammograms for three catalysts obtained upon annealing to 300 °C (orange lines). The main CO_ad_ stripping peak centered at ~0.7 V occurs on all three investigated electrodes. On the Pt@GC_ox_ and Pt@Cr electrodes, a pre-peak can be observed from 0.25 V to about 0.6 V. In contrast, on the Pt@Ni electrode, besides the pre-peak, a small anodic peak appears at ~0.4 V, which can be attributed to the partial oxidation of the CO_ad_ monolayer, i.e., the weakly adsorbed CO [17,47] at Ni sites [48]. As early as 0.25 V, these Ni domains can activate water molecules and generate Ni(oxy)hydroxides on the surface, which react with CO adsorbed at neighboring Pt sites. The presence of Ni(oxy)hydroxides at the surface was confirmed by XPS [41,49,50]. Thus, this peak indicates Ni’s migration from the underlying substrate to the surface due to the annealing process. Numerous investigations have been performed on Pt low-index single crystals, demonstrating that the (110) plane is the most active in the main oxidation region, having the onset potential at more negative values related to (111) and (100) planes [11,51]. Considering this, after annealing, the positive shift observed on Pt@Cr electrodes can be attributed to the increase in (111) and (100) surface domains. In the case of Pt@Ni, it is impossible to conclude since the thermal treatment has also induced the surface composition changes due to Ni migration to the surface; thus, the observed negative shift can also be attributed to the oxidation of CO at Ni sites.

### 3.4. Methanol Electro-Oxidation

**Figure 4:**
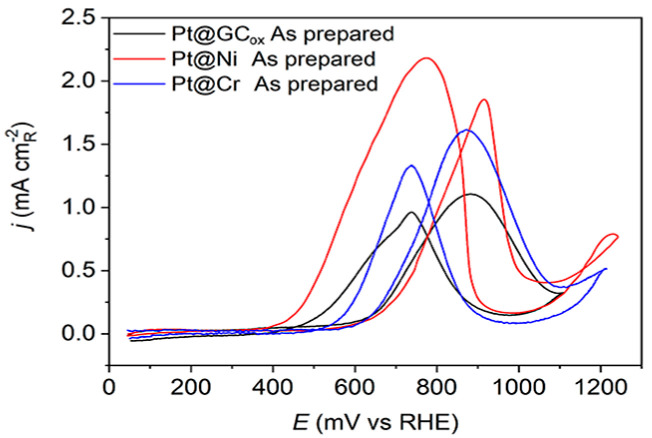
Cyclic voltammograms for the oxidation of 0.5 M CH_3_OH on as-prepared Pt@GC_ox_ (black curve), Pt@Ni (red curve), and Pt@Cr (blue curve) catalysts in 0.1 M HClO_4_. Sweep rate: 50 mV s^−1^.

The methanol oxidation currents on Pt(hkl) at low potentials rely on the single-crystal orientation, demonstrating the reaction’s structural sensitivity. The oxidation of methanol initiates on the (111) plane, then proceeds on the (110) plane, and finally occurs on the (100) plane. This sequence follows the adsorption of OH groups [18]. These data suggest that the initiation of methanol oxidation is correlated to the commencement of OH adsorption [52]. The results obtained using as-prepared Pt thin-film electrodes deposited on different supports, presented in Figure 4, reveal a consistent pattern of methanol oxidation behavior. The positive scans indicate a high activity level, suggesting that all examined catalysts can efficiently adsorb methanol due to sufficient adjacent adsorption sites. Nevertheless, these electrodes cannot adsorb the same quantity of OH^−^ anions. All three Pt@S surfaces have a similar overall voltammetric profile. Still, their highest activity levels varied significantly, which could be due to the substrate’s characteristics or the underlying presence of specific crystal domains. The reaction is initiated, at approximately the same potentials, on Pt@Cr and Pt@GC_ox_ catalysts (E_in_~0.6 V) followed by the Pt@Ni catalyst (E_in_~0.65 V). The reaction occurs predominantly on the surfaces covered by reversible OH_ad_ species, which shows relatively rapid kinetics. The current maximum occurs at peak potentials when the reaction rate of methanol dehydrogenation [53], Equation (1),

CH_3_OH → CO_ad_ + 4H^+^ + 4e^−^ (1)

is finely balanced with the reaction rate of oxidation of dehydrogenation products (CO_ad_) with OH_ad_ species in a Langmuir–Hinshelwood-type reaction, as in Equation (2):

CO_ad_ + OH_ad_ → CO_2_ + H^+^ + e^−^ (2)

The reaction rate described by Equation (2) relies on CO and OH^−^ species coverage. Since θ_CO_ + θ_OH_ ≤ 1, high θ_OH_ or θ_CO_ will reduce the number of Pt sites that are available for the adsorption of methanol or OH^−^ anions. As a result, the rate of reaction will be slow. Hence, it is evident that the well-balanced coadsorption of methanol and water at low potentials would enhance the Pt catalyst’s efficiency. Regardless of their support, two prominent oxidation peaks were recorded on all three electrodes, one at 0.895 V–0.905 V in the forward scan and one at ~0.75 V in the backward scan direction. It is evident that surface structure noticeably influences the relative intensity of both peaks. On the Pt@GC_ox_ and Pt@Cr samples, the oxidation peak in the forward scan has a higher current density than in the backward scan. In contrast, on Pt@Ni, the peak in the backward scan has a higher current density than in the forward scan direction.

**Figure 5:**
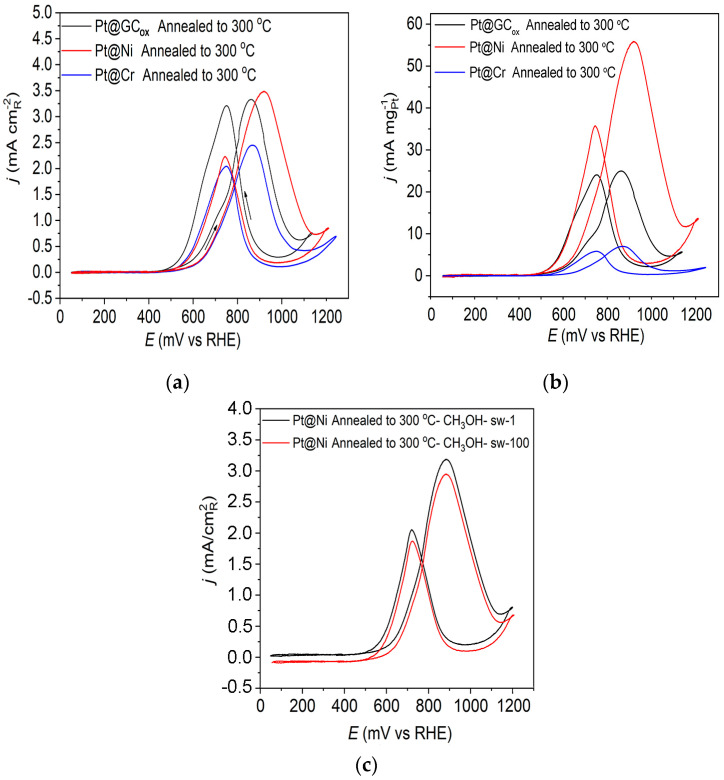
Cyclic voltammograms for the oxidation of 0.5 M CH_3_OH on Pt@GC_ox_, Pt@Ni, and Pt@Cr catalysts annealed to 300 °C in 0.1 M HClO_4_; (**a**) The current density normalized with respect to the active surface area of the catalysts; (**b**) mass-specific current density, and (**c**) stability. Sweep rate: 50 mV s^−1^.

Figure 5a displays the methanol oxidation reaction for the three catalysts obtained upon annealing to 300 °C in 0.1 M HClO_4_. These findings indicate some notable differences in the obtained voltammograms when using Pt catalysts on a different substrate. The reaction initiates at comparatively low potential values (~0.5 V) compared to the onset of the reaction for the as-prepared Pt@S electrodes, which is around 0.6 V, indicating improved catalysts activity (Figure 4). Upon annealing the Pt@GC_ox_ electrode in a reductive atmosphere, it was observed that both peaks in the forward and backward scan exhibited nearly identical characteristics. On the Pt@Cr electrode, the forward scan oxidation peak has a slightly higher current density compared to the oxidation peak in the backward scan. However, on the Pt@Ni catalyst, the peak in the positive scanning direction has a significantly higher current density value than in the negative scan direction. It was concluded that the oxidation peak observed during the reverse scan was not associated with the oxidation of the remaining intermediate, but it most likely originates from the oxidation of methanol on the Pt surface covered with oxygenated species [54]. The ratio between the forward oxidation peak current (If) and backward oxidation peak current (Ib), If/Ib, is not associated with the degree of CO tolerance. Instead, it is associated with oxophilicity.

The obtained mass-specific current densities at Pt@S catalysts annealed to 300 °C are displayed in Figure 5b. Pt@Ni is the best catalyst (considering the amount of precious metal consumed) reaching, at E~0.905 V, a mass-specific current density that is larger by one order of magnitude and ~2 times than that obtained on Pt@Cr and Pt@GC_ox_ catalysts, respectively. The observed behavior is attributed to the significant contribution of smaller Pt particles, the greater specific surface area, and the lack of agglomerates at the Pt@Ni electrode. On Pt@Cr, on the other hand, quite the opposite is observed. Therefore, this catalyst has the lowest mass activity. It should be noted that all catalysts contain approximately the same amount of Pt.

In order to examine the stability of the catalyst, we used prolonged cycling, which showed that in addition to being the most active catalyst, Pt@Ni annealed to 300 °C is also extremely stable. The results showed that there is practically no poisoning of the catalyst after 100 potential cycles, as shown in Figure 5c.

## 4. Conclusions

Catalysts in the form of a thin film present a potential replacement for bulk single crystals, as the nature of their geometry significantly lowers the precious metal content and provides a surface that is susceptible to ordering upon annealing, driven by a tendency to expose the structure with the lowest surface energy. It is widely accepted that surface structure has a significant influence on electrocatalyst activity. Controlled heat treatment is an important technique for inducing surface atom reorganization, thus completely changing the catalyst characteristics. This contribution has demonstrated a novel approach of working with thin films with controllable surface structures. Furthermore, in addition to (111), the creation of the (100) and (110) surface domains could be controlled as well. This represents a major advancement in the creation of commercial catalysts.

By using a carefully selected combination of techniques including AFM, selective Bi adsorption, oxidation of ammonia, and CO_ad_ electro-oxidation, we were able to provide insight into the alteration of the morphology of PtS (S being GC, Ni, Cr) thin-film catalysts.

The substrate structure does not epitaxially force Pt as it was deposited over amorphous (GC) and polycrystalline metal (Ni and Cr) support. However, surface faceting has been achieved by annealing the electrode, which promotes surface diffusion, flattens defects, changes the fraction of crystal planes (100), and encourages the growth of the lowest energy facets, such as (111) sites. AFM data revealed that even a mild annealing temperature of 300 °C was sufficient to induce diffusion across the surface and grain boundary migrations that resulted in coalescence and particle growth.

By examining the obtained results of methanol oxidation on Pt thin films electrochemically deposited over these supports (activated GC, Ni, and Cr) and correlating them with the model system surfaces, i.e., Pt single crystals, the effect of thermal annealing under regulated conditions (temperature and atmosphere) can be associated with the enhanced activity of the Pt@S catalyst. Thus, the thermal treatment applied turned out to be crucial for obtaining superior activity for methanol electro-oxidation.

## Figures and Tables

**Table 1:** The fraction of ordered domains (111) for as-prepared and annealed Pt@GC_ox_, Pt@Ni, and Pt@Cr.

Sample	Pt@GC_ox_	Pt@Ni	Pt@Cr
As prepared	24	8.0	8.6
Annealed to 300 °C	55	24	40

## Data Availability

The data presented in this study are available on request from the corresponding author or co-authors. The data are not publicly available.

## References

[1] Carrette L., Friedrich K.A., Stimming U. (2001). Fuel Cells-Fundamentals and Applications. Fuel Cells.

[2] Frelink T., Visscher W., van Veen J.A.R. (1995). Particle Size Effect of Carbon-Supported Platinum Catalysts for the Electrooxidation of Methanol. J. Electroanal. Chem..

[3] Parsons R., VanderNoot T. (1988). The Oxidation of Small Organic Molecules. A Survey of Recent Fuel Cell Related Research. J. Electroanal. Chem..

[4] Gasteiger H.A., Markovic N., Ross P.N., Cairns E.J. (1994). Temperature-Dependent Methanol Electro-Oxidation on Well-Characterized Pt-Ru Alloys. J. Electrochem. Soc..

[5] Bockris J.O., Conway B.E., White R.E., Bockris J.O., Conway B.E., White R.E. (1992). Modern Aspects of Electrochemistry.

[6] Watanabe M., Motoo S. (1975). Electrocatalysis by Ad-Atoms: Part II. Enhancement of the Oxidation of Methanol on Platinum by Ruthenium Ad-Atoms. J. Electroanal. Chem. Interfacial Electrochem..

[7] de Sá M.H., Moreira C.S., Pinto A.M.F.R., Oliveira V.B. (2022). Recent Advances in the Development of Nanocatalysts for Direct Methanol Fuel Cells. Energies.

[8] Tian H., Yu Y., Wang Q., Li J., Rao P., Li R., Du Y., Jia C., Luo J., Deng P. (2021). Recent Advances in Two-Dimensional Pt Based Electrocatalysts for Methanol Oxidation Reaction. Int. J. Hydrogen Energy.

[9] Iwasita T. (2002). Electrocatalysis of Methanol Oxidation. Electrochim. Acta.

[10] Wieckowski A., Wieckowski A. (1999). Andrew Hamnett Mechanism of Methanol Electro-Oxidation. Interfacial Electrochemistry Theory: Experiment, and Applications.

[11] Marković N.M., Ross P.N. (2002). Surface Science Studies of Model Fuel Cell Electrocatalysts. Surf. Sci. Rep..

[12] Vielstich W., Lamm A., Hubert A. (2003). Gasteiger. Handbook of Fuel Cells: Fundamentals, Technology, Applications.

[13] Strmcnik D.S., Tripkovic D.V., Van Der Vliet D., Chang K.C., Komanicky V., You H., Karapetrov G., Greeley J.P., Stamenkovic V.R., Marković N.M. (2008). Unique Activity of Platinum Adislands in the CO Electrooxidation Reaction. J. Am. Chem. Soc..

[14] Marković N.M., Schmidt T.J., Grgur B.N., Gasteiger H.A., Behm R.J., Ross P.N. (1999). Effect of Temperature on Surface Processes at the Pt(111)—Liquid Interface: Hydrogen Adsorption, Oxide Formation, and CO Oxidation. J. Phys. Chem. B.

[15] Mekazni D.S., Arán-Ais R.M., Ferre-Vilaplana A., Herrero E. (2022). Why Methanol Electro-Oxidation on Platinum in Water Takes Place Only in the Presence of Adsorbed OH. ACS Catal..

[16] Herrero E., Franaszczuk K., Wieckowski A. (1994). Electrochemistry of Methanol at Low Index Crystal Planes of Platinum. An Integrated Voltammetric and Chronoamperometric Study. J. Phys. Chem..

[17] Tripković A.V., Gojković S.L., Popović K.D., Lović J.D. (2006). Methanol Oxidation at Platinum Electrodes in Acid Solution: Comparison between Model and Real Catalysts. J. Serbian Chem. Soc..

[18] Xia X.H., Iwasita T., Ge F., Vielstich W. (1996). Structural Effects and Reactivity in Methanol Oxidation on Polycrystalline and Single Crystal Platinum. Electrochim. Acta.

[19] Cuesta A., Couto A., Rincón A., Pérez M.C., López-Cudero A., Gutiérrez C. (2006). Potential Dependence of the Saturation CO Coverage of Pt Electrodes: The Origin of the Pre-Peak in CO-Stripping Voltammograms. Part 3: Pt(Poly). J. Electroanal. Chem..

[20] Housmans T.H.M., Wonders A.H., Koper M.T.M. (2006). Structure Sensitivity of Methanol Electrooxidation Pathways on Platinum: An on-Line Electrochemical Mass Spectrometry Study. J. Phys. Chem. B.

[21] Sharma S., Pollet B.G. (2012). Support Materials for PEMFC and DMFC Electrocatalysts—A Review. J. Power Sources.

[22] Chalk S.G., Miller J.F. (2006). Key Challenges and Recent Progress in Batteries, Fuel Cells, and Hydrogen Storage for Clean Energy Systems. J. Power Sources.

[23] Maillard F., Bonnefont A., Micoud F. (2011). An EC-FTIR Study on the Catalytic Role of Pt in Carbon Corrosion. Electrochem. Commun..

[24] Ji Y., Cho Y.I., Jeon Y., Lee C., Park D.-H., Shul Y.-G. (2017). Design of Active Pt on TiO_2_ Based Nanofibrous Cathode for Superior PEMFC Performance and Durability at High Temperature. Appl. Catal. B Environ..

[25] Liu Z.Y., Zhang J.L., Yu P.T., Zhang J.X., Makharia R., More K.L., Stach E.A. (2010). Transmission Electron Microscopy Observation of Corrosion Behaviors of Platinized Carbon Blacks under Thermal and Electrochemical Conditions. J. Electrochem. Soc..

[26] Cerri I., Nagami T., Davies J., Mormiche C., Vecoven A., Hayden B. (2013). Innovative Catalyst Supports to Address Fuel Cell Stack Durability. Int. J. Hydrogen Energy.

[27] Kaur A., Kaur G., Singh P.P., Kaushal S. (2021). Supported Bimetallic Nanoparticles as Anode Catalysts for Direct Methanol Fuel Cells: A Review. Int. J. Hydrogen Energy.

[28] Hrnjić A., Kamšek A.R., Bijelić L., Logar A., Maselj N., Smiljanić M., Trputec J., Vovk N., Pavko L., Ruiz-Zepeda F. (2024). Metal-Support Interaction between Titanium Oxynitride and Pt Nanoparticles Enables Efficient Low-Pt-Loaded High-Performance Electrodes at Relevant Oxygen Reduction Reaction Current Densities. ACS Catal..

[29] Fu Q., Dong J., Li H., Xiao J., Yang B., Zhang B., Bai Y., Song T., Zhang R., Gao L. (2020). Reaction-Induced Strong Metal-Support Interactions between Metals and Inert Boron Nitride Nanosheets. J. Am. Chem. Soc..

[30] Pourebrahimi S., Pirooz M., Ahmadi S., Kazemeini M., Vafajoo L. (2023). Nanoengineering of Metal-Based Electrocatalysts for Carbon Dioxide (CO_2_) Reduction: A Critical Review. Mater. Today Phys..

[31] Hanief N., Lang C.I., Bucher R., Topic M. (2012). Phase Transformations and Surface Characterization of the Platinumchromium Coated System. J. S. Afr. Inst. Min. Metall..

[32] Beermann V., Gocyla M., Kühl S., Padgett E., Schmies H., Goerlin M., Erini N., Shviro M., Heggen M., Dunin-Borkowski R.E. (2017). Tuning the Electrocatalytic Oxygen Reduction Reaction Activity and Stability of Shape-Controlled Pt-Ni Nanoparticles by Thermal Annealing -Elucidating the Surface Atomic Structural and Compositional Changes. J. Am. Chem. Soc..

[33] Hoseini S.J., Bahrami M., Samadi Fard Z., Fatemeh Hashemi Fard S., Roushani M., Agahi B.H., Hashemi Fath R., Sarmoor S.S. (2018). Designing of Some Platinum or Palladium-Based Nanoalloys as Effective Electrocatalysts for Methanol Oxidation Reaction. Int. J. Hydrogen Energy.

[34] Rodríguez P., Herrero E., Solla-Gullón J., Vidal-Iglesias F.J., Aldaz A., Feliu J.M. (2005). Specific Surface Reactions for Identification of Platinum Surface Domains: Surface Characterization and Electrocatalytic Tests. Electrochim. Acta.

[35] Papadimitriou S., Tegou A., Pavlidou E., Armyanov S., Valova E., Kokkinidis G., Sotiropoulos S. (2008). Preparation and Characterisation of Platinum- And Gold-Coated Copper, Iron, Cobalt and Nickel Deposits on Glassy Carbon Substrates. Electrochim. Acta.

[36] Bogdanovskaya V.A., Tarasevich M.R., Reznikova L.A., Kuznetsova L.N. (2010). Composition, Surface Segregation, and Electrochemical Properties of Binary PtM/C (M = Co, Ni, Cr) Catalysts. Russ. J. Electrochem..

[37] Urchaga P., Baranton S., Coutanceau C., Jerkiewicz G. (2012). Electro-Oxidation of CO Chem on Pt Nanosurfaces: Solution of the Peak Multiplicity Puzzle. Langmuir.

[38] Solla-Gullón J., Vidal-Iglesias F.J., Herrero E., Feliu J.M., Aldaz A. (2006). CO Monolayer Oxidation on Semi-Spherical and Preferentially Oriented (100) and (111) Platinum Nanoparticles. Electrochem. Commun..

[39] Pajić M.N.K., Stevanović S.I., Radmilović V.V., Rogan J.R., Radmilović V.R., Gojković S.L., Jovanović V.M. (2016). Pt/C Nanocatalysts for Methanol Electrooxidation Prepared by Water-in-Oil Microemulsion Method. J. Solid State Electrochem..

[40] Koper M.T.M. (2011). Structure Sensitivity and Nanoscale Effects in Electrocatalysis. Nanoscale.

[41] Tripković D.V., Milošević D.L., Stevanović S.I., Popović K.D., Jovanović V.M., Lopes P.P., Martins P.F.B.D., Stamenković V.R., Strmčnik D. (2024). Design of Advanced Thin-Film Catalysts for Electrooxidation of Formic Acid. ACS Catal..

[42] Tripković D.V., Popović K.D., Jovanović V.M., Nogueira J.A., Varela H., Lopes P.P., Strmcnik D., Stamenkovic V.R., Markovic N.M. (2019). Tuning of Catalytic Properties for Electrooxidation of Small Organic Molecules on Pt-Based Thin Films via Controlled Thermal Treatment. J. Catal..

[43] Mayrhofer K.J.J., Arenz M., Blizanac B.B., Stamenkovic V., Ross P.N., Markovic N.M. (2005). CO Surface Electrochemistry on Pt-Nanoparticles: A Selective Review. Electrochim. Acta.

[44] Maillard F., Schreier S., Hanzlik M., Savinova E.R., Weinkauf S., Stimming U. (2005). Influence of Particle Agglomeration on the Catalytic Activity of Carbon-Supported Pt Nanoparticles in CO Monolayer Oxidation. Phys. Chem. Chem. Phys..

[45] Chen Y., Shi J., Chen S. (2015). Small Molecule (CO, H_2_) Electro Oxidation as an Electrochemical Tool for Characterization of Ni@Pt/C with Different Pt Coverages. J. Phys. Chem. C.

[46] Antolini E., Salgado J.R.C., Santos L.G.R.A., Garcia G., Ticianelli E.A., Pastor E., Gonzalez E.R. (2006). Carbon Supported Pt-Cr Alloys as Oxygen-Reduction Catalysts for Direct Methanol Fuel Cells. J. Appl. Electrochem..

[47] Tripković A.V., Popović K.D., Lović J.D., Jovanović V.M., Kowal A. (2004). Methanol Oxidation at Platinum Electrodes in Alkaline Solution: Comparison between Supported Catalysts and Model Systems. J. Electroanal. Chem..

[48] Chatenet M., Faure R., Soldo-Olivier Y. (2005). Nickel-Underpotential Deposition on Pt(1 1 0) in Sulphate-Containing Media. J. Electroanal. Chem..

[49] Koolen C.D., Oveisi E., Zhang J., Li M., Safonova O.V., Pedersen J.K., Rossmeisl J., Luo W., Züttel A. (2024). Low-Temperature Non-Equilibrium Synthesis of Anisotropic Multimetallic Nanosurface Alloys for Electrochemical CO_2_ Reduction. Nat. Synth..

[50] Campbell C.T. (1997). Ultrathin Metal Films and Particles on Oxide Surfaces: Structural, Electronic and Chemisorptive Properties. Surf. Sci. Rep..

[51] Lebedeva N.P., Rodes A., Feliu J.M., Koper M.T.M., Van Santen R.A. (2002). Role of Crystalline Defects in Electrocatalysis: CO Adsorption and Oxidation on Stepped Platinum Electrodes as Studied by in Situ Infrared Spectroscopy. J. Phys. Chem. B.

[52] Chung D.Y., Lee K.J., Sung Y.E. (2016). Methanol Electro-Oxidation on the Pt Surface: Revisiting the Cyclic Voltammetry Interpretation. J. Phys. Chem. C.

[53] Yu R., Zhang Y., Deng S., Zhu R., Zhang S., Zhang J., Zhao Y., Xia Z. (2024). Platinum Alloys for Methanol Oxidation Electrocatalysis: Reaction Mechanism and Rational Design of Catalysts with Exceptional Activity and Stability. Catalysts.

[54] Wang X., Wang H., Wang R., Wang Q., Lei Z. (2012). Carbon-Supported Platinum-Decorated Nickel Nanoparticles for Enhanced Methanol Oxidation in Acid Media. J. Solid State Electrochem..

